# Proteome changes of *Caenorhabditis elegans *upon a *Staphylococcus aureus *infection

**DOI:** 10.1186/1745-6150-5-11

**Published:** 2010-02-17

**Authors:** Annelies Bogaerts, Isabel Beets, Liesbet Temmerman, Liliane Schoofs, Peter Verleyen

**Affiliations:** 1 Research Group of Functional Genomics and Proteomics, KU Leuven, Naamsestraat 59, 3000 Leuven, Belgium

## Abstract

**Background:**

The success of invertebrates throughout evolution is an excellent illustration of the efficiency of their defence strategies. *Caenorhabditis elegans *has proven to be an appropriate model for transcriptome studies of host-pathogen interactions. The aim of this paper is to complement this knowledge by investigating the worm's response to a *Staphylococcus aureus *infection through a 2-dimensional differential proteomics approach.

**Results:**

Different types of growth media in combination with either *E. coli *OP50 or *Staphylococcus aureus *were tested for an effect on the worm's lifespan. LB agar was chosen and *C. elegans *samples were collected 1 h, 4 h, 8 h and 24 h post *S. aureus *infection or *E. coli *incubation. Proteomics analyses resulted in the identification of 130 spots corresponding to a total of 108 differentially expressed proteins.

**Conclusions:**

Exploring four time-points discloses a dynamic insight of the reaction against a gram-positive infection at the level of the whole organism. The remarkable upregulation after 8 h and 24 h of many enzymes involved in the citric acid cycle might illustrate the cost of fighting off an infection. Intriguing is the downregulation of chaperone molecules, which are presumed to serve a protective role. A comparison with a similar experiment in which *C. elegans *was infected with the gram-negative *Aeromonas hydrophila *reveals that merely 9% of the identified spots, some of which even exhibiting an opposite regulation, are present in both studies. Hence, our findings emphasise the complexity and pathogen-specificity of the worm's immune response and form a firm basis for future functional research.

**Reviewers:**

This article was reviewed by Itai Yanai, Dieter Wolf and Torben Luebke (nominated by Walter Lutz).

## Background

The nematode *Caenorhabditis elegans *lives in the soil, where it encounters and feeds on a wide variety of bacteria and fungi. However, not all encounters result solely in a nutritional benefit. Some of these microbes are known pathogens, capable of infecting and even killing *C. elegans*. Therefore, the worm has developed many ways to cope with the harmful effects of an infection. The activation of a physiological immune response is accompanied by high costs in terms of energy. Thus preventing an encounter with a pathogen would seem to present the best option.

In fact, *C. elegans *has been known to exhibit avoidance behaviour towards different types of pathogenic micro-organisms. By means of chemo and mechanoreceptors they can sense and distinguish between bacterial compounds in their surroundings [1,2]. The full genetics underlying this behavioural trait are yet to be elucidated. Briefly, it is known that *tol-1*, a homologue of the *Drosophila *Toll-receptor, plays a role. Mutants of this receptor are defective in avoiding pathogenic bacteria such as *Serratia marcescens *[3]. Furthermore, activation of the insulin-like receptor (ILR) pathway suppresses this ethological response [4]. Recent studies also indicate that there is a neural regulation of innate immunity as well as pathogen avoidance behaviour. G protein coupled receptors (GPCR's) may be part of a neural circuit, that integrates signals from infected areas or from pathogens and which subsequently converts these signals in an appropriate defence response [5,6]. More specific, animals deficient in NPR-1, a homologue of the neuropeptide Y receptor in mammals, show a decrease in pathogen avoidance and a decreased innate immune response [7,8].

If getting away from the threat is no option, the first-line defence of the worm is a physical barrier. This barrier consists of a cuticle which forms the interface between the worm and the environment, and the pharynx, that breaks up all oral intake of micro-organisms. If a pathogen succeeds in overcoming these barriers, it will be detected by so-called pathogen recognition molecules. The detection results in the activation and interplay of the seven main signalling cascades: the p38 mitogen-activated protein kinase (MAPK), the insulin-like receptor (ILR), the Toll-like receptor (TLR), a transforming growth factor-b (TGF-b), the programmed cell death (PCD), the extracellular signal-regulated kinase (ERK) and a c-Jun N-terminal kinase (JNK). Recently, Kaplan *et al*. 2009 suggested that *C. elegans *can attract its bacterial food and is even capable of partially regulating the virulence of bacterial pathogens by inhibiting specific Quorum Sensing systems [9].

This current knowledge about the *C. elegans *immune system has predominately been gathered through forward and reverse genetic studies and is fully discussed in many recent reviews [10-14]. Although these genetic studies are indispensable, with this paper we aimed at approaching the immunity question from another level, more specific the protein level. Which proteins function in the immune response of *C. elegans*? Are they subjected to post-translational modifications (PTMs)? Which proteins are differentially expressed (up or down) after an infection with *Staphylococcus aureus*? To answer these questions we used two-dimensional difference gel electrophoresis (2D-DIGE), a fluorescence-based method that increases the power of the proteomics technique by allowing two different protein samples tagged with two distinct fluorescent dyes to be run on the same gel. Such an approach enables a rapid screening for differences in protein profiles between naive and immune-challenged *C. elegans*. Samples were taken at 4 time-points after infection in order to follow the expression profile of potentially important immune proteins in time. As there is only a poor correlation between mRNA levels and the proteins they code for [15,16], the outcome of this proteomics study can provide valuable new insights.

Studying bacterial pathogenesis is not an easy task. Prior to the proteomics analysis we verified the growth condition of *S. aureus *and the effect on the lifespan of *C. elegans *as there have been indications that the virulence and even the mode of infection of a bacterium is influenced by the type of growth medium they are cultivated on. *Pseudomonas aeruginosa *PA14, for instance, can kill *C. elegans *in two distinct ways depending on the amount of nutrients in their growth medium. In low salt medium, PA14 kills in a slow manner through accumulation of bacteria in the worm intestines. As a consequence, worms die over a period of 2-3 days [17]. In a high salt and rich medium, worms are killed within 2-4 hours by the production of diffusible toxins (fast-killing) [18]. Another *Pseudomonas aeruginosa *strain PA01, kills *C. elegans *in yet another way. When grown on brain-heart infusion media, worms exposed to this strain become paralysed and die within 4 hours [19]. To establish the best possible experimental design, we tested 3 different media (nematode growth medium (NGM), LB agar and TS agar) in survival assays in which lifespan of worms grown on standard *E. coli *OP50 was compared to lifespan of worms fed with *Staphylococcus aureus*. This to make sure that *S. aureus *definitely does, and our control *E. coli *OP50 does not, act as a pathogen. Indeed, analogous to transcriptome experiments [20], this *E. coli *strain was chosen as a control because after thousands of generations, *C. elegans *is well adopted to this species [21].

*Staphylococcus aureus *is a versatile and wide-spread gram-positive bacterium. Besides living as a commensal on human skin, nose and throat, this bacterium can cause a range of illnesses from minor skin infections to life-threatening diseases such as meningitis, endocarditis and toxic shock syndrome (TSS). It is one of the most common causes of nosocomial infections, often the source of postsurgical wound infections. Furthermore, *S. aureus *has the ability to develop resistance against many common antibiotics and can form slow-growing subpopulations (small colony variants) that show a heightened or attenuated virulence [22]. This bacterium was amongst the first models applied in *C. elegans *immunity research [23,24]. With this paper we demonstrate the potential of a differential proteomics approach to further dissect the worm's response on a *S. aureus *infection.

## Results

### Survival assays

**Figure 1:**
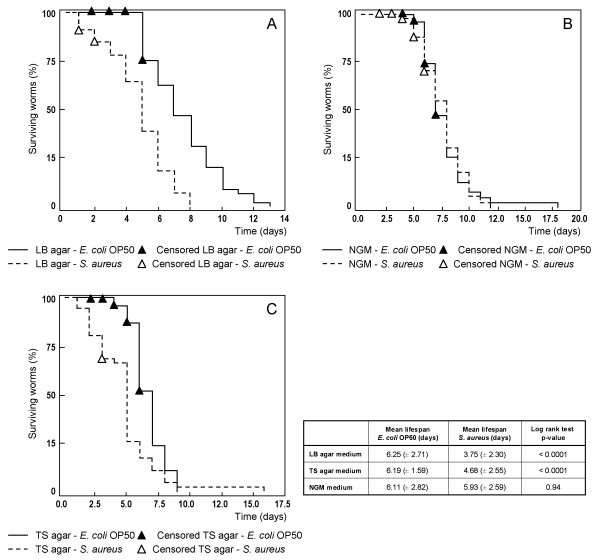
**Kaplan- Meier plot for the survival of *C. elegans *on different media and bacteria**. The Kaplan-Meier plot for *C. elegans *fed with either *E. coli *OP50 or *Staphylococcus aureus *on a LB agar (A), NGM (B) or TS agar (C) medium. The results of the paired log rank test as well as the mean lifespan (± standard deviation) of worms grown on both bacteria are indicated. P-value < 0.001 was adopted as statistically significant.

A survival assay was conducted to determine the effect of a *S. aureus *infection on the lifespan of *C. elegans*. As the type of growth medium may have an influence on the virulence of a bacterium, we tested three different growth media: NGM, LB agar and TS agar. This to make sure that our control *E. coli *OP50 does not, whilst our pathogen *S. aureus *does, shorten lifespan of worms. A log rank test was performed to search for significant differences in survival between the two conditions. P-value < 0.001 was accepted as statistically significant. Worms that escaped from the plates were included in the analysis as "censored" data. The Kaplan-Meier plots for *C. elegans *fed with either *E. coli *OP50 or *S. aureus *are shown in Figure 1. Table 1 compiles the resulting χ^2^-statistics and p-value for each comparison. *S. aureus *displays a negative impact on the survival of *C. elegans*, if cultivated on LB agar or TS agar plates. When the bacteria are grown on NGM medium, there is no significant difference in survival between worms on *E. coli *OP50 and worms fed with *S. aureus*. Furthermore, the survival of worms on *E. coli *OP50 on a TS agar medium significantly differs from standard cultivating conditions (*E. coli *OP50 on NGM). Thus, when grown on TS agar, our control *E. coli *OP50 also has a negative influence on lifespan of *C. elegans*. Hence, we choose LB agar plates to perform further infections and sample preparation for 2D-DIGE experiments.

**Table 1:** Statistical results of the survival assays.

	χ^2^- statistics	p-value
***E. coli *OP50 versus *S. aureus***		
LB agar medium	35.1154	<0.0001
TS agar medium	23.5703	<0.0001
NGM medium	0.0054	0.9413
		
***S. aureus***		
LB agar medium - TS agar medium	0.0000	0.9995
LB agar medium - NGM medium	39.6801	<0.0001
TS agar medium - NGM medium	28.8096	<0.0001
		
***E. coli *OP50**		
LB agar medium - TS agar medium	7.6000	0.0058
LB agar medium - NGM medium	0.2212	0.6382
TS agar medium - NGM medium	11.9542	0.0005

Paired log-rank test to evaluate the effect of growth medium and bacterium type on the lifespan of *C. elegans*. The resulting χ^2 ^and p-values indicate a significantly shorter lifespan when grown on *S. aureus *on LB or TS agar and *E. coli *on TS agar.

### 2D-DIGE

**Figure 2:**
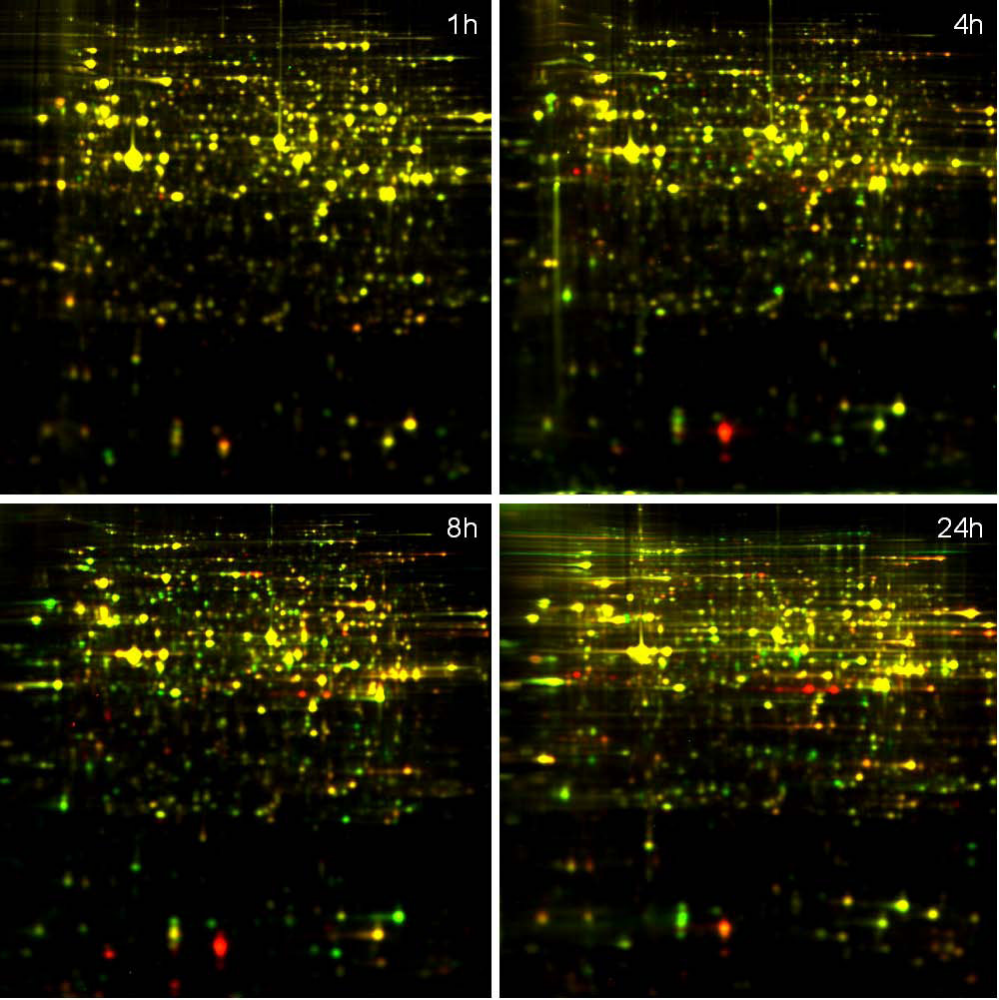
**DIGE images of *C. elegans *at different time-points upon infection**. The false-colored protein expression profile of *C. elegans *1, 4, 8 and 24 hours after infection with *S. aureus*. Propyl-Cy3 labeled proteins of worms infected with *S. aureus *are colored in red and methyl-Cy5 labeled proteins of worms in standard conditions are colored in green.

**Figure 3:**
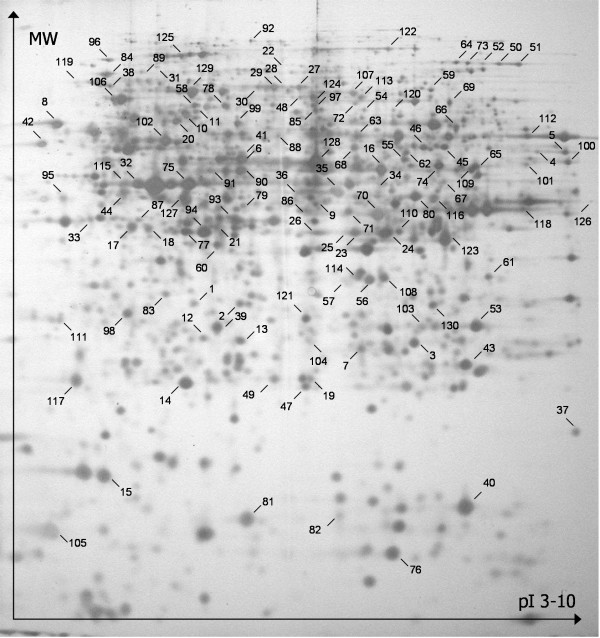
**Identified spots on the 2D proteome map of infected *C. elegans***. A silver stained 2-DE map of the immune-induced proteome of *Caenorhabditis elegans*. The map is restricted to proteins with a molecular mass ranging from 10-200 kDa and a pI from 3-10. The numbered spots were all identified (see Additional file 1).

By means of two-dimensional differential gel electrophoresis and mass spectrometry, the proteome of *C. elegans *was investigated 1, 4, 8 and 24 hours after infection with *Staphylococcus aureus *NCTC 8325. From the survival assay, we learnt that the mean lifespan of worms infected with *S. aureus *is 3.75 days. Thus, to minimalise the number of spots on the gel image caused by degradation of proteins (from dying worms), the last time-point in our study was 1 day after infection. At this point the worms are expected to still be relatively fit. Figure 2 shows a false-coloured DIGE image at all 4 time-points. It is clear that the number of differentially expressed (reddish and greenish) spots increases in function of time, which is reflected by the number of identified differential proteins (see Additional file 1). The number of spots matched on a single gel varied between 2050 and 3200 among the 24 gels. With the Decyder 7.0 software we selected the spots of which the expression level alters with more than 25% and with a corrected p-value < 0.05. From the first experiment, one hour after *S. aureus *infection, we identified 25 differentially expressed proteins of which 7 were up- and 18 were downregulated. Experiment 2 yielded 44 proteins, 15 up- and 29 downregulated. Experiment 3, 8 hours after microbe challenging, resulted in a list of 95 identified proteins, 48 up- and 47 downregulated. Finally, the DIGE experiment 24 hours after initial infection, gave 98 identifications of which 53 up- and 43 downregulated proteins. In total, 130 spots were identified as several proteins were identified in more than one experiment. These 130 spots correspond to only 108 differentially expressed proteins. This discrepancy is most likely the result of several post-translational modifications (PTM) or different splice variants of the same protein. Protein R11A5.4, for example, was found in spot 27 to 30. These 4 proteins spots are all orientated adjacent to each other in a train-like formation (Figure 3), probably caused by phosphorylation of the original R11A5.4. This Figure 3 represents a silver stained map of the infected *C. elegans *proteome on which all identified proteins are indicated. Additional file 1 lists all identified proteins and the degree of up- or downregulation of their expression level at the time-points on which these are statistically significant. Hypothetical *C. elegans *proteins were blasted in search for human orthologues. If present with an E-value < e-30, the name of the human orthologue was listed in italics in Additional file 1.

The data from this publication are accessible from the World-2DPAGE database http://world-2dpage.expasy.org/repository/database=0020. Details concerning the applied materials and methods (MIAPE) can be accessed via http://miapegeldb.expasy.org/experiment/70/

## Discussion

Exploring the capability of *S. aureus *to infect and kill *C. elegans*, three distinct growth media were tested: NGM, LB agar and TSA agar. The result is bipartite. First of all, there is a negative interaction between host and pathogen when grown on a LB or TS agar medium. In 2003 Sifri *et al*. reported a reduction in lifespan of *C. elegans *when fed with *S. aureus *on a TS agar medium. Further research showed the accumulation of bacteria in the worm intestines [24]. Although the exact molecular mechanisms of infection are not fully elucidated, it is known that part of this infection is manifested through toxins. Also virulence factors, involved in *S. aureus *pathogenesis in mammals, play a crucial role. They consist of extracellularly secreted factors, such as the cytolytic alpha-hemolysin and the V-8 protease, and regulatory genes that control the expression of other virulence factors such as *agr *and the *sarA *locus [24,25]. On a NGM medium, there is no significant difference between lifespan of worms feeding on *S. aureus *or *E. coli *OP50. Thus, the virulence of this pathogen clearly depends on the type of growth medium. McNamara and Proctor (2000) have already shown that the expression of virulence factors in *S. aureus *decreases when an insufficient amount of energy is available [26]. This may explain why this bacterium has no effect on the survival of worms when cultivated on a minimal medium such as NGM. Furthermore, it is possible that specific components, that are required for the production of virulence factors, are missing in the NGM medium. The second conclusion that can be made from this preliminary experiment is that *E. coli *OP50 is slightly pathogenic when grown on TS agar. This phenomenon has also been observed for e. g. brain heart infusion medium on which *E. coli *OP50 has a deleterious effect on *C. elegans *[23,27,28]. Based on this dual conclusion, for our further infection and DIGE experiments, we opted to work with LB agar medium on which *S. aureus *does and *E. coli *OP50 does not behave as a pathogen for *C. elegans*.

Previous forward and reverse genetic studies have unravelled many components of the immune signalling cascades. In addition, potential recognition molecules and antimicrobial peptides have been put forward. However, knowledge about the exact changes that occur specifically at protein level of an immune-challenged worm remains limited. After a successful pilot study, using differential proteomics and mass spectrometry to investigate the proteome of *C. elegans *infected with *Aeromonas hydrophila *[29], we expanded our search to the response on a gram-positive pathogen with the same technique. As an immune response is a dynamic process, four time-points were chosen based on the survival assay on which a DIGE experiment was performed. For instance, the expression of spot 23 gradually increases in time. However, some proteins are characterized by a strong upregulation at only one time-point. This could suggest a specific role for this protein at that specific moment. Our data illustrate that an analysis at only one time-point is not sufficient to get a complete picture of the changes that occur after an infection. Moreover, the expression profile of a protein can give a hint towards its function. For instance, proteins that are characterized by an increased expression level in the initial phase of infection may act as recognition or signalling molecules which lead to the actual immune response. Furthermore, proteins with a gradually increasing expression are likely to play an effector role.

**Figure 4:**
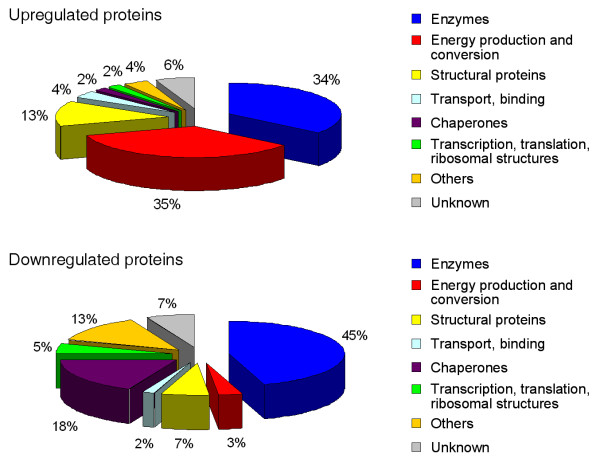
**Functional categories of the differentially expressed proteins**. Pie charts representing the functional categories of the up- and downregulated *C. elegans *proteins identified in this study after infection with *S. aureus*.

In this study a total of 130 spots corresponding to 108 differentially expressed proteins were identified. These proteins can be subdivided into categories based on their functional properties (Figure 4) and illustrate how the infection is anticipated at the level of the whole organism at different time-points. The most elaborate group of differentially expressed proteins is made up of enzymes involved in carbohydrate, lipid, amino acid and nucleotide metabolism. Notable is the upregulation of numerous proteins involved in energy production and conversion. We identified no less than 8 enzymes (ACO-2, F53F4.10, W02F12.5, FUM-1, IDH-2, SDHA-1, CTS-1, F23B12.5) that are part of the citric acid cycle which might reflect the need for extra energy whilst fighting off an infection. A group of proteins well represented in the downregulated section are proteins involved in stress responses such as heat shock proteins. Two smaller categories are structural and transport proteins. We found proteins involved in transcription and translation such as subunits of ribosomal proteins. The last but perhaps most interesting group consists of hypothetical proteins for which no functional information is available yet. For example, hypothetical protein F17C11.9, was found significantly downregulated in two distinct spots, 8 and 24 hours after infection (Additional file 1, Figure 3, spots 79 and 80). Our paper is the first indication for its potential role in the immune response of *C. elegans*.

### Alcohol dehydrogenase

The most strongly upregulated protein (Additional file 1, spot 23) in this study was an alcohol dehydrogenase (SODH-1). Albeit a 1200% upregulation 24 hours after infection, the lifespan of a *sodh-1 *deficient mutant (RB2114) did not differ from that of a wildtype in our survival assay with *S. aureus *(data not shown). Sorbitol dehydrogenase belongs to the superfamily of medium-chain dehydrogenases/reductases (MDR) and shows a high sequence similarity to the tetrameric alcohol dehydrogenase found in yeast [30]. Alcohol dehydrogenases facilitate the interconversion between potentially toxic alcohols and aldehydes or ketons. In yeast, these enzymes play an important role in the fermentation process: the formation of ethanol from acetaldehyde under anaerobic conditions. The expression of this enzyme is heightened in stress-resistant dauer larvae [31]. Furthermore, in worms grown in an axenic medium, high amounts of this protein were observed [32]. Because alcohol dehydrogenase converts glyceraldehyde into glycerol, Castelein et al. (2008) postulate a function for this enzyme in lipid metabolism and glyceroneogenese [32]. Given the resemblance to the yeast alcohol dehydrogenase, this may suggest an anaerobic shift in metabolism of infected worms. The involvement of SODH-1 in the defence responses of *C. elegans *remains obscure. However, an altered expression of *sodh-1 *has also been observed in other immune studies [33,34,29]. Striking is the fact that this protein is upregulated after infection with a gram-positive bacterium and downregulated in case of a gram-negative infection. This could indicate a different metabolic response to both types of bacteria or could have a more specific immune-related meaning. The expression of *sodh-1 *is positively regulated by the DAF-16 transcription factor [35]. DAF-16 is part of the ILR pathway, one of the 8 immune signalling cascades in *C. elegans*. Also in other organisms, alcohol dehydrogenase has been linked to immunity. For instance, the expression of this enzyme strongly increases after an immune-challenge by means of LPS (lipopolysaccharide) in fruit fly larvae [36]. This suggests a more specific function for alcohol dehydrogenase in invertebrate host defence.

### Acyl-CoA dehydrogenase

Acyl-CoA dehydrogenases catalyze the initial step of fatty acid beta-oxidation. This mitochondrial enzyme was identified from three spots, upregulated after 4 hours (spot 34) and downregulated after 8 and 24 hours (spot 35, 36). Thus, different forms of this protein may have distinct functions. Transcriptome studies of the immune response of *C. elegans *against the gram-negative bacterium *Pseudomonas aeruginosa *also report a downregulation of *acdh-1 *[20,34]. Furthermore, a decrease in expression level was observed in fasted animals [37] and as a response to heavy metals [34]. The reduced expression of this enzyme under pernicious living conditions may indicate an alteration in the lipid metabolism of the worm. However, since recent studies have shown that lipids, like polyunsaturated fatty acids, form an important group of signal molecules that might regulate the defence mechanisms in *C. elegans *[38], enzymes involved in lipid metabolism may play a more specific role in immunity. This hypothesis is supported by the immune-induction of other enzymes involved in beta-oxidation of fatty acids such as enoyl-CoA hydratase (*ech-6*) and *maoc-1 *(Additional file 1).

### Comparison to the gram-negative infected proteome

When comparing our dataset to the list of differentially expressed proteins after infection with the gram-negative *Aeromonas hydrophila *[29], certain general patterns can be discerned. In both studies we observe the induction of oxidative stress proteins such as glutathione S-transferases, proteins involved in necrosis such as vacuolar H+ ATPases (predominately in the last time-points of infection) and galactose binding lectins. Via binding to sugar residues on the surface of pathogens, galectins are believed to play a role as pathogen recognition molecules [39]. We identified LEC-1 and LEC-2. As their expression levels remained elevated at the last measured time-point, galectins may even possess an antimicrobial effector function. A more surprising finding was the downregulation of a lipid binding protein (LBP-6). In other immune studies, these lipid binding proteins generally have a positive association with defense responses, as in our previous study where we found an upregulated *lbp-1 *and *lbp-7*. Next, a tendency in both studies is the diminished expression of chaperones which contradicts the general assumption that these proteins could have a protective role in case of an infection. On the other hand, other downregulations of *C. elegans *heat shock proteins are known such as *hsp-16.4 *upon infection with *Serratia marcescens *[20]. Calreticulin is an endoplasmatic reticulum resident chaperone that, together with calnexin, is responsible for the correct folding and maturation of many newly synthesized proteins [40]. Lee *et al*. (2006) postulated the existence in *C. elegans *of an alternative chaperone machinery in case of a calreticulin/calnexin deficiency. The identified proteins HSP-3, HSP-4 and PDI-2 are presumed to be part of this compensating chaperone mechanism [41]. Thus, CRT-1 on the one hand and HSP-3, HSP-4 and PDI-2 on the other hand, are most likely involved in similar processes. In fact, these four proteins are found downregulated at least at one time point after infection with *S. aureus *(Additional file 1). In case of stress or inefficient functioning of the calreticulin/calnexin system, unfolded proteins may accumulate in the endoplasmatic reticulum. To restore normal ER functions the so-called 'unfolded protein response' (UPR) will be activated. In *C. elegans*, this signalling pathway has been linked to innate immunity [42]. Activation of UPR genes offers protection against bacterial pore-forming toxins [43]. However, in our paper the target genes of UPR are down-regulated. Could this be the result of an evasion strategy of the pathogen? Why *C. elegans *decreases the expression of chaperones that might protect the worm against the harmful effects of a gram-positive or gram-negative infection remains unclear.

Only 16 spots corresponding to 14 different proteins were found in both infection experiments (Additional file 2). Most of these common proteins show a similar alteration in expression level. Nevertheless, there are two exceptions: ALH-8 and SODH-1. Whilst *alh-8*, an aldehyde dehydrogenase, is upregulated in case of a gram-negative infection, we observe a decrease in *alh-8 *expression for a gram-positive infection and vice versa for *sodh-1*. This fact plus the limited overlap in differentially expressed proteins between both studies underlines the pathogen-specific nature of the immune response of *C. elegans*.

## Conclusions

An effort was made to enrich the knowledge about *C. elegans *immunity by performing a differential proteomics study on worms infected with *S. aureus*.

Prior to the DIGE experiments we established the best possible experimental design, by measuring lifespan of *C. elegans *on *S. aureus *and *E. coli *OP50 for three different growth media in survival assays. We observed that the virulence of a bacterium clearly depends on the cultivating conditions. Based on this experiment, we chose to work with the LB agar medium on which *S. aureus *does and *E. coli *OP50 does not behave as a pathogen.

Proteomics at four different time points on infected worms resulted in a list of potentially important immune proteins. Some of these proteins were already linked to immunity, but we were able to identify an additional group of proteins with unknown function. These proteins form an interesting lead to gain further functional insight in the worm's immune response.

Recently, DIGE has proven to be a successful tool to investigate the worm's interaction with a gram-negative and a gram-positive pathogen. Although some general patterns can be observed, the overlap between both datasets comprises merely 9%. In addition, some proteins show a different expression pattern depending on the type of infecting bacterium, accentuating the complexity of the immune response of *C. elegans*.

We hope that our data provide a strong stimulus to further explore *C. elegans*' immune response at the level of the proteome.

## Methods

### Nematode and bacterial strains

*Caenorhabditis elegans*, N2 Bristol wildtype strain, was cultured by standard methods on Luria Bertani agar plates at 20°C [44]. The wildtype strain was obtained from the Caenorhabditis Genetics Centre (CGC). Bacterial strains used in this study were *E. coli *OP50 as standard food source and *Staphylococcus aureus *NCTC 8325 as pathogen. *S. aureus *NCTC 8325 was a kind gift from Dr. Costi D. Sifri (University of Virginia). *E. coli *OP50 was again obtained from the CGC.

### Survival assays

A synchronous population of *C. elegans *was established by isolating eggs from adult worms through bleaching [45]. After hatching, L1 worms were transferred to LB agar plates seeded with *E. coli *OP50 and allowed to grow at 20°C until they reached the L4 stadium. Worms were washed for several times with a 0.1 M NaCl solution. Using the COPAS biosorter (Union Biometrica), 15 worms were placed on plates containing either *E. coli *OP50 or *S. aureus *bacteria. For each condition 5 plates and thus 75 worms were followed. The L4 stadium was defined as t = 0 and *C. elegans *were maintained at 24°C. Worms were daily transferred to fresh plates, dead animals were counted and removed. Worms that escaped from the plates were censored. Statistical analysis was performed using STAT 9.1 software. To evaluate differences between conditions, a log rank test was carried out. P-value < 0.001 was adopted as statistically significant. This survival experiment was performed with nematode growth medium (NGM), Tryptic Soy agar (TS agar) or Luria Bertani agar (LB agar) plates.

### Infecting worms and sample preparation

*E. coli *OP50 bacteria were grown in Luria Bertani and *S. aureus *in Tryptic Soy medium overnight at 37°C. 100-200 μl of liquid culture was spread on LB agar plates and incubated overnight at 37°C. Synchronous L4 worms were obtained as described in the previous section. Next, the worms were collected and washed five times in a 0.1 M NaCl solution to remove bacteria. Thereafter, the falcon tubes were centrifuged at 13000 rpm for 4 min at 4°C. The supernatant was removed and the concentrated suspension of worms was equally spread on LB agar plates containing either *E. coli *OP50 as control or *S. aureus *as pathogen. After 1, 4, 8 and 24 hours an extract was made of worms in both conditions and for each condition three biologically independent samples were taken. Worms were collected in falcon tubes with a 0.1 M NaCl solution and washed two times. To establish an adequate separation of live worms and unwanted organic material, a sucrose floatation procedure was applied [45]. Thereafter, the worms were washed 6-7 times with a 0.1 M NaCl solution. To reduce the presence of bacterial spots on the gel image, worms were left to digest their remaining intestinal content by gently shaking the falcon tubes for one hour at room temperature. The NaCl solution was removed and the worm pellet was suspended in twice the volume of a lysis solution containing 7 M urea, 2 M thiourea, 4% CHAPS, 40 mM Tris, 1% DTT and Complete protease inhibitor (Roche). The suspensions were homogenised and put on ice. Subsequently, the suspensions were sonicated five times for 5 sec and put on ice during the intervals. Then, the samples were centrifuged for 12 min at 13000 rpm and 4°C. Supernatants were desalted using the PlusOne Mini Dialysis Kit (GE Healthcare). Protein concentration was determined by the method of Bradford [46]. For each condition, 75 μg of proteins was labelled with fluorescent dyes Cy3 or Cy5 (GE Healthcare). Both a forward (immune-challenged proteins labelled with Cy5, control proteins with Cy3) and reverse (immune-challenged with Cy3 and control with Cy5) labelling were performed. For each time-point, an internal standard, consisting of 37,5 μg of each sample, was labelled with Cy2.

### 2D electrophoresis

2D electrophoresis was run in darkness. For isoelectric focusing (IEF) Immobiline pH 3-10 NL Drystrips (24 cm, GE Healthcare) were rehydrated overnight in Destreak solution and 0.5% IPG buffer (GE Healthcare). 75 μg of test sample labelled with Cy3 or Cy5, 75 μg of control sample labelled with Cy5 or Cy3 and 75 μg of internal standard labelled with Cy2 were mixed and loaded in cups. IEF was performed with the Ettan IPGphor Manifold (GE Healthcare) at 20°C and 50 μA per IPGstrip; 3 h at 150 V, 3 h at 300 V, 6 h at 1000 V and 8000 V until 50,000 Vh. Strips were stored at -80°C. Prior to SDS-PAGE, IPG strips were immersed twice for 15 min in equilibration buffer (6 M urea, 50 mM Tris-Cl (pH 8.8), 30% glycerol and 2% SDS). Respectively DTT (1% w/v) and iodoacetamide (4% w/v) were added. Equilibrated strips were placed on top of a 1.5 mm SDS-polyacrylamide gel (11.5% T; 2.6% C) and run in the Ettan Daltsix (GE Healthcare) at 20°C; 1 h at 600 V, 10 mA/gel and 10 W; overnight at 600 V, 14 mA/gel and 15 W.

### Gel imaging and image analysis

Gel images were obtained with the Ettan DIGE imager (GE Healthcare). Briefly, the gels were illuminated with the specific excitation wavelengths of Cy3 (540/25 nm), Cy5 (635/30 nm) and Cy2 (480/30 nm). For image and statistical analysis, the DeCyder software version 7.0 (GE Healthcare) was used. Spot exclusion filter was set to 30000 counts and the false discovery rate (FDR) application was used to filter out false positives.

### Trypsin digestion of protein spots and mass spectrometry

After imaging and analysis, spots of interest were excised either manually or by means of a spotpicker (GE Healthcare). For manual excision, spots were visualized on the gel through silver staining according to Shevchenko *et al*. [47]. Spots were excised with a sterile scalpel and silver ions were removed prior to digestion. To each spot, 25 μl of 30 mM potassium ferricyanide and 25 μl of 100 mM sodium thiosulfate were added. The gel pieces were vortexed until the brown colour disappeared and rinsed with Milli-Q water. The following steps are identical with the procedure for the spotpicker. We dehydrated the gel pieces three times with 50 μl of CH_3_CN. Next, gel pieces were reswollen during 10 min with 50 μl of 100 mM ammonium bicarbonate and dehydrated again with CH_3_CN for 10 min. The last two steps were repeated and spots were dried. For enzymatic digestion, gel pieces were covered with 25 μl of a digestion buffer (50 mM ammonium bicarbonate and 5 mM CaCl_2_) containing 6 ng/μl trypsin (Promega) and incubated on ice for 45 min. Following enzymatic digestion overnight at 37°C, the resultant peptides were extracted in three steps of each 30 min: once with 80 μl of 50 mM ammonium bicarbonate and twice with 80 μl of 50% CH_3_CN and 5% formic acid (FA). The samples were analyzed by MALDI-TOF MS (matrix assisted laser desorption/ionization time-of-flight mass spectrometer) (Bruker Reflex) as described in Vierstraete *et al*. [48]. Proteins were identified through Peptide Mass Fingerprinting (PMF) using Mascot (Matrix Science). One missed cleavage per peptide was allowed and a mass tolerance between 0.3 and 0.1 Da was used in all searches. Carbamidomethylation was set as fixed modification, oxidation (M) as variable. We searched the general NCBI database with either all entries or specifically *Caenorhabditis elegans*.

## Competing interests

The authors declare that they have no competing interests.

## Authors' contributions

AB designed the study, performed data collection and drafted the manuscript. IB & LT contributed to data collection and analysis. LS & PV co-conceived the research and contributed to the manuscript draft.

## Reviewers' comments

### Reviewer's report 1

Dr. Itai Yanai, Technion Israel Institute of Technology, Haifa, Israel.

### Reviewer comments

In this manuscript Bogaerts and colleagues, examine the proteomic effect of a bacterial pathogen upon the nematode *C. elegans*. For each of four time points following infection with the gram-positive pathogen *Staphylococcus aureus*, the authors sample the proteome using differential 2D gene electrophoresis and match spots with proteins using mass spectrometry. They identify 108 proteins which were differentially expressed in the infected strain and attempt to gain insight into the molecular nature of the response. The most represented group of differentiated proteins comprises enzymes involved in energy production and conversion indicating the high energy cost of fighting off an infection. Many of the differentially expressed proteins are known to participate in immune responses supporting the efficacy of this method.

Analysis of the proteomic response was mainly at the level of examining annotations. However, it would have also been interesting to examine the interaction between the different proteins found in the assay. The protein-protein interaction network of the proteins found to be differentially regulated in the response to infection might expose some interesting aspects of the immune response and its energy cost. Further it could be of importance to test the hypothesis that protein variants themselves may exhibit a differential response to infection. For example, the protein R11A5.4 of which two variants show up-regulation while two other variants show down-regulation with time of infection. Another example for differential regulation is seen in the case of ACGH-1. Elaboration of the differential response of protein variants may provide insights into their role in the fight against an infection.

### Authors' response

The suggested experiments are perfect examples of what we and hopefully other groups (after reading our paper) might do to further elaborate the precise function of the significantly differentially expressed proteins we found in our study.

Thus, in our view, our paper offers sound data, complementary to the large amount of data at the level of the transcriptome, which are the basis for further research and which can trigger many follow-up papers.

### Reviewer comments

The authors mention a separate experiment and analysis for the *C. elegans *response to the gram-negative pathogen *Aeromonas hydrophila*. It would be interesting to compare between these two responses. Such an evaluation could reveal the general mechanisms of an immune response invoked by any infectious host and the more specific components of responses towards particular pathogens.

### Authors' response

The other two reviewers raised this point as well.

First of all, we agree that all the cited references should be available. Therefore, we added this manuscript as an additional file [29]. Please, note that this manuscript is under review for a different journal, and thus does not make "officially" part of our submission to Biology Direct.

To facilitate the comparison between both papers, we added a third (Additional file 2), representing all proteins which are (significantly) differential in both studies.

We agree that similarities and differences between both studies might hint at universal and specific responses against pathogens. However, before drawing such conclusions we would prefer to analyze additional time points and certainly several other pathogens with a similar approach. Thus we did not largely change the paragraph summarizing the differences between gram-positive and negative bacterial infections of *C. elegans*.

### Reviewer comments

Further analysis could be directed towards testing specific hypotheses by mutant analysis. As mentioned by the authors it would be of interest to compare the response of a wildtype to a mutant immune-challenged *C. elegans *strain. The list of potentially important immune proteins could be used to find mutant strains and compare their proteomic response to infection to the one of the wildtype strain.

### Authors' response

Again, these are valuable suggestions, for future experiments. For example, we found that both lec-1 and sodh-1 deficient mutants do not show a reduced survival compared to infected wild type worms. It would of course be interesting to repeat the proteomics approach in case of such an equally long living mutant to search for compensating mechanisms, but this would double the work and cost and we suggest this to be yet another challenge for follow-up papers.

We assume that the above proposed experiments are not major concerns against our paper, but are ideas for further follow-up studies, illustrating the potential of our data to trigger/provoke future investigations as stated in the conclusion of this review?

### Reviewer comments

One minor concern involves the discussion of alcohol dehydrogenase's possible involvement in the infection response. We find the suggestion of an anaerobic shift in metabolism of infected worms as a rather unlikely explanation for the strong up-regulation of this protein during infection. The second mentioned option of the role of alcohol dehydrogenase in lipid metabolism and glyceroneogenesis is much more reasonable and fits well with the concept of the infectious energy cost.

### Authors' response

We admit that the first explanation is farfetched, especially compared to the much more reasonable second option. Therefore, we removed that particular sentence and adjusted the text.

### Reviewer comments

Overall, this paper contributes to the effort of indentifying the molecular signature of the response to infection and constitutes a firm basis for future in-depth investigation of this important field.

### Reviewer's report 2

Dr. Dieter Wolf, Burnham Institute for Medical Research, La Jolla, CA, United States

### Reviewer comments

Using 2D-PAGE, a method of limited resolving power, the authors have undertaken a proteomic analysis of *C. elegans *challenged with *S. aureus *for 1, 4, 8, and 24 h. By quantitative image analysis and mass fingerprinting, they identified ~100 proteins that were differentially regulated (minimum change of 25%). The proteins can be sorted into various functional categories, and the categories are discussed with respect to their possible biological significance.

The main strength of the study is that it provides an initial proteomic signature of worms incubated with *S. aureus*. Significant weaknesses include the limited depth of the analysis (maybe 500 proteins surveyed on the gels), the lack of validation of at least some of the expression changes by immunoblotting, the apparent lack of biological replicates analyzed by 2D-PAGE (uncertainty the expression changes are reproducible), and the absence of any biological validation of the expression changes observed. With respect to the latter point, it would have been interesting to know whether manipulating the levels of some of the altered proteins would lead to increased or decreased sensitivity to *S. aureus*.

Secondly, a large portion of the discussion concentrates on data that are not shown in this manuscript, but in another manuscript that is pending publication elsewhere. Since the present study is less rich in data than desirable, the authors might want to consider combining the datasets on gram-positive and -negative bacteria for a single publication either in Biology Direct or elsewhere.

### Authors' response

#### Limited depth of the analysis

We feel that the 2D-DIGE approach is underestimated.

Of course one is studying the most abundant proteins. Nevertheless, the number of matched spots varied between 2050 to 3200 among the 24 gels (containing 48 samples). We added theses figures to the manuscript.

2D-DIGE has successfully been applied in numerous disease studies. It has been proven to be a very elegant technique for biomarker or pathogen discovery.

Besides this gel-based method, also gel-free proteomic techniques are available. One can discuss about which proteomic method is best suited for this type of research. However, gel-based and gel-free techniques each have their advantages and drawbacks [49]. They actually complement each others limitations.

The main reason we opted to use 2D-DIGE is the potential of this technique to give a hint about possible post translational modifications of a protein. For example acdh-1 is upregulated in spot 34 and downregulated in spot 35 and 36.

Thus a simple modification (e.g. phosphorylation), which can be observed by 2D-DIGE, can alter the activity of a given protein activation. Hence a equal total amount of all the 'isoforms' of a o protein can in fact contain different fractions of (in)active 'isoforms'.

Furthermore, a similar amount of mRNA between two conditions might still represent an altered and thus involved protein. This illustrates, among others, how our proteomics approach can complement the various transcriptome studies on *C. elegans*.

To facilitate the appreciation of the value of our 2D-DIGE data and to enhance interpretation of the manuscript, as requested by another reviewer, we added links to a MIAPE document and an interactive 2D-repository where we deposited our data in great detail. We adjusted the text.

#### Lack of validation

Because of the above example and because discrepancies between levels of mRNA and amount of its protein do exist in general, we believe that validation at the level of the transcriptome would add very little information.

A validations by means of immunoblotting, on the other hand, is a valuable suggestion we considered. However, we do not agree with the lack of replicates. For each time point, 3 completely different biological replicates were conducted (pooling thousands of worms) for both conditions (*E. coli *versus *S. aureus*) and a dye swap was performed on top. For one time point, all six replicates were run together, reducing technical variability. On top, an internal standard of all samples was added for each time point.

As at least 2050 spots were measured and statistically evaluated, false positives are of course generated. To counter this, we applied a false discovery correction and used the corrected p-values only.

A physiological validation is certainly the challenge for the future. We did only a small first attempt by performing physiological tests on mutant strains or strains of which expression of specific proteins is compromised by RNAi. We specifically performed survival assays for two such proteins: a sodh-1 mutant and the lec-1 mutant. Unfortunately we did not see any significant differences in lifespan between wildtype and mutant worms. On the other hand and from an evolutionary point of view, these results make sense. It would be illogical that an immune response completely depends on one gene. Thus, the interplay of differential proteins of our proteomics analyses is important. Moreover, one should realise that our data represent not only "pure immune proteins" but signatures of the reaction against a gram-positive infection on the level of the whole organism. It would of course be interesting to repeat the proteomics approach in case of such an equally long living mutant to search for compensating mechanisms, but this would double the work and cost and we suggest this to be yet another challenge for follow-up papers.

In conclusion, because of the experimental set-up (biological replicates, dye swap, and corrected p-values) we believe that additional confirmation is not necessary an sich. In our view, our paper offers an initial picture of the changes that occur at protein level upon infection, in order to provide a reference work. This study resulted in a quite extensive list of proteins which are potentially important in the immune system of *C. elegans*. Further analyzing in depth the effect and/or physiological role of the significantly differentially expressed proteins of interest is the challenge for follow-up papers. In such a context, validation of every protein found differentially expressed, can be a start or control to further explore the meaning of this change upon infection.

#### Comparison with a submitted manuscript

We agree that all the cited references should be available. Therefore, we added this manuscript as an additional file. Please, note that this manuscript is under review for a different journal, and thus does not make "officially" part of our submission to Biology Direct.

### Reviewer's report 3

Dr. Torben Luebke, University of Göttingen, Germany. Nominated by dr. Walter Lutz, University of Göttingen, Germany.

Bogaerts and coworkers describe a comparative analysis of the proteomes of *Caenorhapditis elegans *after *Staphylococcus aureus *infection and non-pathogenic *E. coli *cultivation. First of all the authors show that various growth conditions (full growth media vs. minimum growth media) have a considerable influence on the pathogenesis of the worms. The authors finally decide to perform the proteome analyses on worms cultivated on LB media with the appropriate bacteria due to the unambiguous negative influence on the worms' life span upon *S. aureus *infection. The real proteome analyses were realized by using a 2-dimensional fluorescence difference gel electrophoresis (2D-DIGE) at several time points during infection and a reasonable number of 108 differentially expressed proteins were identified and categorized regarding their function.

However, the study is suffering from various shortcomings which have to be dispelled before publication:

Major comments:

Introduction

1) Please explain why gram-negative *E. coli *rather than a gram-positive non-pathologic bacteria was chosen for control purposes (in order to reduce parameter)?

### Authors' response

This is a relevant question. We decided to choose *E. coli *strain OP50 in the control experiment, not (only) to allow a comparison with the other proteomics experiment we refer to, but mainly because the OP50 strain is thé standard culture medium to grow wild type *C. elegans *(N2) on. This implies 1) that other studies also incorporate OP50 as a control. For example, the reference work of Wong et al., entitled: Genome-wide investigation reveals pathogen-specific and shared signatures in the response of *Caenorhabditis elegans *to infection [20]. 2) that after several thousands of generations *C. elegans *is completely adapted to *E. coli *OP50 as demonstrated by Roeder et al. [21]. In other words, even grown on a different bacterium, N2 worms will still express antimicrobial components specifically targeting *E. coli*. If one's focus is the specific response of N2 worms against virulence factors of a given pathogen (which is not our scoop), the best option might be to compare virulent and non-virulent strains of this microbe of interest.

We added a sentence to the introduction to justify OP50 as a relevant control for our approach.

### Reviewer comments

2) Please specify the known immune response mechanisms of *C. elegans *in the introduction section. So far, mainly the avoidance behaviour is shortly described

### Authors' response

We admit that a limited part of the introduction/background is devoted to the actual immune response and associated molecular mechanisms. Our aim was to write an original introduction focussed on the host-pathogen interaction as the general scheme of innate immunity is described already so often in many excellent reviews, several of which are cited in our paper.

Nevertheless, we added the names of the major pathways involved in the worm's innate immunity to the introduction.

### Reviewer comments

Results

3) The result sections of 1.5 pages are cut down to a minimum so that many details - especially of the 2D-DIGE - are lacking. I would appreciate to get information about the total spot numbers of the appropriate gels and description of individual proteins spots regarding expression levels over the time. The entire Figure 2 including four DIGE gels is reported in as less as three sentences.

I would suggest fusing the result section with the discussion section.

### Authors' response

Obviously, we tried to write the results section as concise as possible and focussed on the results section. Many of the requested details can be inferred from the summarizing Additional file 1. As this table is presented in the supplemental data section (due to its length), we added some more details to the results section. In addition, we added the link to the expasy site (password protected until publication) where our data are presented according to the standards of the Human Proteome Organisation.

Note that Figure 2 is rather a necessary illustration of the quality of our 2D-DIGE data and of the tendency of an increasingly differential proteome in correlation with a longer exposure to the pathogen. This paper comprises 24 high quality gels, the least number of matched spots on a single gel was 2050, the maximum 3200.

### Reviewer comments

4) It does not become clear at what time point 130 spots corresponding to 108 proteins are differentially expressed.

### Authors' response

We addressed this comment in the previous reply.

### Reviewer comments

5) What proteins underwent post-translational modifications? Could you e.g. also identify integral membrane proteins in the gel-based system?

### Authors' response

We added an example of post-translational modification (PTM) in the rewritten section. In addition, whether or not a given protein is found in the gel close to its predicted pI and MW is an indication for the absence of presence of PTMs. The interactive expasy websites displaying our data (see below comment 3 for the link) allow a straightforward comparison.

The gel-based system is not best suited to study all integral membrane proteins. A protein with for example a single transmembrane region and a large intra and/or extracullular sequence has a good change to be separated and identified. Receptors with 7 TMRs will not be detected.

### Reviewer comments

6) How many proteins (total numbers) were upregulated or downregulated? Please conduct the reader punctually through the additional file 1 and give comments on the chosen proteins e.g. sodh-1 in spot 23. Or what protein(s) was identified in the large red protein spot at the bottom of the gel.

### Authors' response

Part of this comment was already addressed below comment 3. We hope that the section we added to the results will guide the reader through our large amount of data. Next, it is not clear to us what exactly is meant by "chosen proteins"? sodh-1 is already extensively discussed in our text. The large red protein spot at the bottom of the gel was not identified because it was not differential. This spot is a very rare and extreme example of preferential Cydye labelling. In other words, this spot not only appeared red on the forward but also on the reversed labelled gels. Thus, thanks to our proper scientific set-up ("dye swap") this spot is not judged as differential. In addition, we admit that other papers often show both a representative reverse and forward labelled image. Thus in a way, as we investigated 4 different time points, we present four times the information of a common DIGE paper.

### Reviewer comments

7) The study misses a validating/confirmation experiment particularly with regard to unexpected downregulation the chaperone/hsp protein family. I would suggest Western blot analyses or - if no antibodies are available - RealTime PCR to get a respective hint on transcriptome level at least.

### Authors' response

In general, we think that checking the transcriptome level is not the best option, because the correlation between the amount of mRNA and the amount of protein or certainly the amount of (in)activated protein (e.g. as a consequence of PTM) does not necessarily correlate. The possibilities to observe different 'states' of proteins is one of the strengths of the applied 2D-DIGE method. In addition, we emphasize that we conducted enough replicates and used corrected p-values to exclude false positives in our set of differential data. Our paper is meant as a complementary approach to all the published data at the level of the transcriptome already published in the field. Also, why additionally validating a particular set of proteins and not all of them? We hope that our data can form the firm basis for future functional analysis focussed on the potentially most interesting differential proteins and that the reviewers can follow this view.

In particular, the downregulation of chaperones seems unexpected at first sight, but this is not a unique case. Whereas most transcriptome studies show an upregulation of most chaperones in case of infected animals, downregulated ones do exist as well (e.g. *hsp-16.41 *is downregulated upon infection with *Serratia marcescens *[20]. At the protein level, we would like to refer to our other 2D-DIGE paper in which HSP-60 is downregulated. Another example is a sHSP which is downregulated in stressed water fleas (unpublished results of a collaborator).

### Reviewer comments

8) The upregulation of the citric acid cycle proteins could be confirmed by simple enzymatic measurements e.g. SDH measurement (sdha-1 is upregulated).

### Authors' response

This comment is already partially addressed above.

Because of the experimental set-up (biological replicates and corrected p-values) we believe that additional confirmation is not necessary *an sich*. In our view, our paper offers sound data which can trigger many follow-up papers, further analyzing in depth the effect and/or physiological role of the significantly differentially expressed proteins of interest. In such a context, validation of every enzyme found differentially expressed, can be a start or control to further explore the meaning of this change upon infection.

### Reviewer comments

9) In order to easily identify spots on Figure 2 (24 h) and Figure 3, MW standards at the y-axis and pH markings at the x-axis should be inserted. I was totally lost when I tried to identify spot 23 in Figure 2.

### Authors' response

We understand this concern. Luckily, this problem is now solved by providing the interactive expasy website referred to in the answer to comment 3.

### Reviewer comments

Discussion section

10) Shortcomings - gel-based system, no receptors.

### Authors' response

Of course both gel-based and gel-free methods have their advantages and shortcomings. In fact, both are complementary, provided enough replicates are conducted. Remarkably, comparative analyses between gel-free and gel-based of the exactly the same sample are rare. In fact, we conducted such an experiment on honeybee hemolymph [49]. To the point, our feeling is that an extensive motivation and/or explanation of the applied technique versus the alternatives are out of the focus of our paper. As a consequence, the paper is perhaps less accessible for researchers who are unfamiliar with proteomics, but that is the choice we wish to make, in the knowledge that countless reviews and technical papers exist on these techniques.

### Reviewer comments

11) The discussion repeatedly refers to a pilote study on *Aeromonas hydrophila*. However, the study is not published yet and was not attached to the manuscript so that this quotation is hardly traceable.

### Authors' response

We agree that all the cited references should be available. Therefore, we added this manuscript as an additional file [29]. Please, note that this manuscript is under review for a different journal, and thus does not make "officially" part of our submission to Biology Direct. To facilitate the comparison between both papers, we added a third supplemental table (Additional file 2), representing all proteins which are (significantly) differential in both studies.

### Reviewer comments

12) It is stated on page 9, last paragraph that the most interesting group consists of hypothetical proteins for which no function is available yet. Please name and specify these proteins or ... do you mean those proteins of the following paragraphs like alcohol dehydrogenase (sodh-1). What is the basis for calculation to receive a 1200% upregulation? In the additional table, a 13fold upregulation is stated.

### Authors' response

We did not mean the proteins of the following paragraphs; their identity is clear, data about their molecular function are available. In stead, we meant the proteins without a clear identity; thus the "hypothetical protein X", as enlisted in Additional file 1. To prevent confusion, we added this sentence to the manuscript:

For example, hypothetical protein F17C11.9, was found significantly downregulated in two distinct spots, 8 and 24 hours after infection (Additional file 1, Figure 3, spots 79 and 80).

The remark about the calculation illustrates how thoroughly our paper was read, but leads perhaps to a more linguistic and/or mathematical discussion? We feel that a 13 fold increase equals a 1200% upregulation. For example, 26 versus 2 is a 13-fold increase (2 × 13 = 26) or an additional 1200% (2+1200 × 2/100 = 26).

### Reviewer comments

13) Are the components of the calreticulin/calnexin system downregulated (if so - please specify in the result section)? The paragraph (page 10) implies that calreticulin/calnexin system is deficient and compensated by hsp-3 etc...! Do the results support this statement?

### Authors' response

13) The Additional file 1 (protein 42) shows that calreticulin (crt-1) is indeed downregulated. Lee et al. found that worms deficient in crt upregulate hsp-3, hsp-4 and pdi-2 [41]. Hence, both protein sets are most likely involved in similar functions. In our data we find all 4 being downregulated in Additional file 1. Stating that this finding supports the data of Lee et al. is perhaps far fetched. Nevertheless, we added two sentences to the manuscript, pointing at this coincidence as suggested by the reviewer.

### Reviewers' comments on the final manuscript

**Reviewer 1 - **Dr. Itai Yanai

Congratulations on the revised manuscript.

**Reviewer 2 - **Dr Dieter Wolf

The revisions and explanations in the rebuttal have clearly improved the manuscript. In particular, the submission of the data to the Expasy database is useful. It is still disappointing, however, that the authors resist the attempt to validate some of their data by immunoblotting. The point here is not the biological validation of specific protein changes; rather this goes to the overall reliability of the dataset. If they could show that the expression differences measured by 2D-DIGE could be replicated in direction and extent for a few protein by immunblotting, this would add a lot of credence to the dataset as a whole. One would assume that some antibodies are available for at least a hand full of the ~100 proteins identified as regulated by infection.

**Reviewer 3 - Dr **Torben Luebke (nominated by Walter Lutz).

The authors responded extensively to my criticism although not always in a fully satisfying manner. Hence I support the acceptance of the manuscript for publication under reserve as follows:

1.) I strongly recommend that at least two proteins (e. g. hsp, sodh or acdh) that are differentially expressed in the DIGE analysis are validated by Western blotting analysis.

2.) Please accentuate and discuss the fact that *S. aureus *is gram-positive while *E. coli *in the control group is gram-negative and thus is not ideal as reference group (although *C. elegans *is well adopted to *E. coli*).

I declare that I have no competing interests in relation to the reviewed manuscript

### Author's response

We do not agree that the technique of gel-based proteomics needs an additional technical confirmation via western blotting, provided that stringent parameters were used for both the determination of a given spot as differential and the identification of a given spot, as we did.

In general, western blotting is of course an excellent tool to start an in depth study of a limited set of proteins, allowing to e.g. closely monitor the levels of a specific protein after several time-points (1 h, 2 h, 3 h...) of infection with different bacteria in a much more cost and time efficient way than 2D-proteomics can ever do.

More specifically, to repeat and confirm 2D-proteomics results (selected by stringent parameters), western blot does not add much and even has its limitations. First of all, the antibody should be specific and validated. Most available antisera are raised against vertebrate proteins, thus their interaction with an orthologous *C. elegans *protein must be confirmed. Next, does the antibody only recognise the protein of interest? In addition, the common 1D blots might not discern the 4 different forms of R11A5.4, we found to be differentially present. Second, like for quantitative PCR, a profound normalisation is necessary. Whereas for qRT-PCR establishing a set of multiple control genes per tissue and condition becomes more and more good practice, this is not (yet) at all the case for western blot studies. This stands in contrast with the situation for 2D-proteomics, where spot intensities are normalised by a majority of similar proteins.

In conclusion, as establishing a set of reliable reference proteins and validating or/and raising antisera for western blot studies would not add much to our present data, we prefer to publish our findings as such in order to enable follow-up studies.

## Supplementary Material

Additional file 1 **Protein identifications (Table 2)**. List of identified proteins that are differentially expressed 1 h, 4 h, 8 h and/or 24 h after infection with *S. aureus*. Proteins in bold were also identified after infection with the gram-negative bacterium *Aeromonas hydrophila *(Bogaerts et al. 2010, ref 29). Protein names in italic represent the human orthologues of the hypothetical *C. elegans *proteins with an E-value < e-30 upon PBLAST. Click here for file

Additional file 2 **Comparison table (Table 3)**. Summary of proteins which are found differentially expressed upon challenge with both *S. aureus *(this paper) and *A. hydrophila *(Bogaerts et. al 2010, ref 29). "+" is up- and "-" is downregulated at least at one time point of the experiment. Proteins in red show an opposite pattern between the two conditions. Click here for file
