# Supplementary material for: Proteome changes of Caenorhabditis elegans upon a Staphylococcus aureus infection
Source: Biol Direct. 2010 Feb 17;5:11. doi: 10.1186/1745-6150-5-11 (PMC2834640; doi:10.1186/1745-6150-5-11)
Supplement: Additional file 1 — Protein identifications (Table 2). List of identified proteins that are differentially expressed 1 h, 4 h, 8 h and/or 24 h after infection with S. aureus. Proteins in bold were also identified after infection with the gram-negative bacterium Aeromonas hydrophila (Bogaerts et al. 2010, ref 29). Protein names in italic represent the human orthologues of the hypothetical C. elegans proteins with an E-value < e-30 upon PBLAST. [file 1745-6150-5-11-S1.DOC]

Table 2: **Protein identifications**. List of identified proteins that are differentially expressed 1h, 4h, 8h and/or 24h after infection with *S. aureus*. Proteins in bold were also identified after infection with the gram-negative bacterium *Aeromonas hydrophila* (Bogaerts et al. submitted). Protein names in italic represent the human orthologs of the hypothetical *C. elegans* proteins with an E-value < e-30 upon PBLAST.

| spot nr | NCBI identification | protein name | Gene name | Theoretical pI | Theoretical Mw (Da) | seq cov (%) | score | Peptides matched | ratio | | | |
| --- | --- | --- | --- | --- | --- | --- | --- | --- | --- | --- | --- | --- |
|  |  |  |  |  |  |  |  |  | 1h | 4h | 8h | 24h |
| **1** | NP_492766.1 | F25H2.10 | rpa-0 | 6.27 | 33870 | 49 | 89 | 14 | 1.42 |  |  | 1.94 |
| 2 | NP_491258.1 | FK506-Binding protein family member (fkb-5) | fkb-5 | 5.80 | 30260 | 61 | 126 | 20 | -1.39 | -1.92 | -1.31 |  |
| 3 | NP_496563.1 | Triose Phosphate Isomerase family member (tpi-1) | tpi-1 | 6.23 | 26672 | 60 | 133 | 13 | 1.34 |  | 1.53 | 1.43 |
| 4 | NP_501862.1 | GABA TransAminase family member (gta-1) | gta-1 | 8.67 | 53364 | 28 | 69 | 14 | 1.28 | 1.43 | 1.47 |  |
| 5 | NP_501862.1 | GABA TransAminase family member (gta-1) | gta-1 | 8.67 | 53364 | 25 | 76 | 12 |  | 1.19 | 1.65 | 1.27 |
| 6 | NP_495900.1 | ENOLase family member (enol-1) | enol-1 | 5.56 | 46759 | 75 | 300 | 31 | -1.98 | -1.52 |  |  |
| 7 | NP_506376.1 | *NADH-Ubiquinone oxidoreductase flavoprotein 2* | F53F4.10 | 6.54 | 26453 | 49 | 81 | 12 | -1.6 |  |  |  |
| 8 | NP_872239.1 | Protein Disulfide Isomerase family member (pdi-2) | pdi-2 | 4.76 | 49265 | 48 | 133 | 22 | -1.56 |  |  | -1.7 |
| 9 | NP_872091.1 | *Adenosine deaminase* | C06G3.5 | 5.79 | 39456 | 43 | 99 | 15 | -1.55 |  |  |  |
| **10** | NP_495134 | Intermediate Filament, B family member (ifb-2) | ifb-2 | 5.41 | 59850 | 43 | 172 | 19 | -1.53 |  | -3.91 | 1.66 |
| 11 | NP_495133.1 | Intermediate Filament, B family member (ifb-2) | ifb-2 | 5.45 | 61688 | 65 | 316 | 42 | -1.36 |  |  | 1.81 |
| 12 | NP_496489.1 | proteasome Regulatory Particle, Non-ATPase-like family member (rpn-12) | rpn-12 | 5.56 | 28777 | 37 | 114 | 13 | -1.47 | -1.64 | -1.69 | -1.8 |
| 13 | NP_497713.1 | *GrpE protein homolog1* | C34C12.8 | 8.37 | 26546 | 46 | 75 | 13 | -1.43 | -1.54 | -1.71 | -1.33 |
| 14 | NP_872052 | PeRoxireDoXin family member (prdx-2) | prdx-2 | 5.53 | 21885 | 58 | 179 | 11 | -1.42 |  | -1.28 | -1.19 |
| 15 | NP_510828.1 | Myosin Light Chain family member (mlc-2)] | mlc-2 | 5.06 | 18648 | 76 | 84 | 13 | -1.4 |  | 1.36 | 2.56 |
| **16** | NP_510002.1 | S-Adenosyl Methionine Synthetase family member (sams-1) | sams-1 | 6.02 | 43840 | 51 | 152 | 19 | -1.38 | -1.54 | -1.81 | -1.48 |
| 17 | NP_001024421.1 | DIsorganized Muscle family member (dim-1) | dim-1 | 5.17 | 35802 | 67 | 154 | 21 | -1.37 | -1.4 |  |  |
| 18 | NP_001024421.1 | DIsorganized Muscle family member (dim-1) | dim-1 | 5.17 | 35802 | 44 | 107 | 13 | -1.24 | -1.26 | -1.39 |  |
| 19 | NP_497892.1 | PeRoxireDoXin family member (prdx-3) | prdx-3 | 6.95 | 25259 | 42 | 90 | 12 | -1.31 |  |  |  |
| **20** | NP_741117.1 | Chaperonin Containing TCP-1 family member (cct-5) | cct-5 | 5.41 | 59654 | 57 | 291 | 39 | -1.28 | -1.28 | -1.4 | -1.49 |
| 21 | NP_508948.2 | hypothetical protein T21F4.1 | T21F4.1 | 5.64 | 36216 | 42 | 89 | 14 | -1.25 |  | -1.93 | -2.01 |
| 22 | NP_494884 | Glutathione S-Transferase family member (gst-8) | gst-8 | 6.16 | 23475 | 23 | 57 | 6 | 3.67 | 6.75 | 5.33 | 2.65 |
| **23** | NP_505991.1 | SOrbitol DeHydrogenase family member (sodh-1) | sodh-1 | 6.07 | 38185 | 59 | 153 | 19 |  | 2.49 | 7.72 | 13.08 |
| **24** | NP_505991 | SOrbitol DeHydrogenase family member (sodh-1) | sodh-1 | 6.07 | 38185 | 31 | 107 | 13 |  | 1.97 | 3.9 | 7.15 |
| 25 | NP_505991.1 | SOrbitol DeHydrogenase family member (sodh-1) | sodh-1 | 6.07 | 38185 | 38 | 104 | 15 |  |  | 6.99 | 7.31 |
| 26 | NP_505991.1 | SOrbitol DeHydrogenase family member (sodh-1) | sodh-1 | 6.07 | 38185 | 42 | 126 | 16 |  |  | 3.3 |  |
| 27 | NP_001021587.1 | *Phosphoenolpyruvate carboxykinase 2 isoform 1* | R11A5.4 | 5.79 | 74012 | 31 | 125 | 22 |  | 2.12 | 3.39 | 2.91 |
| 28 | NP_001021590.1 | *Phosphoenolpyruvate carboxykinase 2 isoform 1* | R11A5.4 | 5.92 | 70105 | 29 | 129 | 18 |  | 1.35 | 1.73 | 2.31 |
| **29** | NP_001021590.1 | *Phosphoenolpyruvate carboxykinase 2 isoform 1* | R11A5.4 | 5.92 | 70105 | 46 | 208 | 31 |  |  | -1.79 | -1.43 |
| **30** | NP_001021587.1 | *Phosphoenolpyruvate carboxykinase 2 isoform 1* | R11A5.4 | 5.79 | 74012 | 37 | 117 | 23 |  |  | -1.51 | -1.25 |
| 31 | NP_001024803.1 | ACTin family member (act-4) | act-4 | 5.56 | 40628 | 26 | 70 | 12 |  | 1.39 | 1.52 | 1.36 |
| 32 | NP_001024803.1 | ACTin family member (act-4) | act-4 | 5.56 | 40628 | 45 | 140 | 22 |  | 1.25 | 1.38 |  |
| 33 | NP_001024803.1 | ACTin family member (act-4) | act-4 | 5.56 | 40628 | 36 | 64 | 12 | 3.09 | 5.38 | 3.97 | 7.63 |
| 34 | NP_491871.1 | Acyl CoA DeHydrogenase family member (acdh-1) | acdh-1 | 6.42 | 47574 | 36 | 122 | 19 |  | 1.34 |  |  |
| 35 | NP_491871.1 | Acyl CoA DeHydrogenase family member (acdh-1) | acdh-1 | 6.42 | 47574 | 46 | 158 | 21 |  |  | -1.98 | -4.86 |
| 36 | NP_491871.1 | Acyl CoA DeHydrogenase family member (acdh-1) | acdh-1 | 6.42 | 47574 | 23 | 95 | 11 |  |  | -1.43 | -3.69 |
| 37 | NP_493624.1 | CYclophyliN family member (cyn-5) | cyn-5 | 8.98 | 21913 | 31 | 61 | 7 |  | -1.8 |  |  |
| 38 | NP_495536.1 | Heat Shock Protein family member (hsp-4) | hsp-4 | 5.03 | 72358 | 44 | 167 | 28 |  | -1.47 | -1.85 |  |
| 39 | NP_496755.1 | DAF-16/FOXO Controlled, germline Tumor affecting family member (dct-18) | dct-18 | 5.69 | 24726 | 67 | 96 | 13 |  | -1.44 | -1.76 | -1.52 |
| 40 | NP_492761.1 | *Nucleoside diphosphate kinase B isoform 1* | F25H2.5 | 6.97 | 17220 | 51 | 83 | 9 |  | -1.43 | -2.03 |  |
| 41 | NP_508026.1 | FK506-Binding protein family member (fkb-6) | fkb-6 | 5.65 | 48315 | 25 | 113 | 20 |  | -1.36 | -1.27 | -1.48 |
| 42 | NP_504575.1 | CalReTiculin family member (crt-1) | crt-1 | 4.59 | 45816 | 36 | 87 | 16 |  | -1.34 | -1.57 | -1.25 |
| 43 | NP_505829.1 | ATP synthase subunit family member (atp-5) | atp-5 | 6.67 | 21841 | 86 | 132 | 16 |  | -1.31 |  |  |
| 44 | NP_504045.1 | hypothetical protein R08E5.3 | R08E5.3 | 5.07 | 41274 | 34 | 84 | 12 |  | -1.29 | -1.56 | -1.46 |
| 45 | NP_498081.2 | ALdehyde deHydrogenase family member (alh-1) | alh-1 | 7.14 | 55309 | 46 | 151 | 23 |  | -1.29 |  | 1.44 |
| 46 | NP_498081.2 | ALdehyde deHydrogenase family member (alh-1) | alh-1 | 7.14 | 55309 | 30 | 129 | 16 |  |  | 1.69 | 1.84 |
| 47 | NP_497116.1 | Glutathione S-Transferase family member (gst-27) | gst-27 | 5.79 | 23261 | 68 | 127 | 15 |  | -1.26 | -1.39 | -1.13 |
| 48 | NP_001040727.1 | hypothetical protein C01F1.3 | C01F1.3 | 5.76 | 71696 | 19 | 67 | 12 |  | -1.26 | -1.37 | 1.29 |
| 49 | NP_497115.1 | Glutathione S-Transferase family member (gst-26) | gst-26 | 5.70 | 23277 | 59 | 96 | 12 |  | -1.25 | -1.5 | -1.17 |
| 50 | NP_741235.1 | ACOnitase family member (aco-2) | aco-2 | 8.16 | 84450 | 42 | 160 | 27 |  |  | 2.82 | 2.01 |
| 51 | NP_741235.1 | ACOnitase family member (aco-2) | aco-2 | 8.16 | 84450 | 21 | 77 | 10 |  |  | 2.79 | 2.03 |
| 52 | NP_741235.1 | ACOnitase family member (aco-2) | aco-2 | 8.40 | 72825 | 21 | 72 | 10 |  |  | 2.66 | 1.98 |
| **53** | NP_499156.1 | Enoyl-CoA Hydratase family member (ech-6) | ech-6 | 8.58 | 31380 | 40 | 122 | 10 |  |  | 2.05 |  |
| 54 | NP_001021412.1 | *Pyruvate kinase isozymes M1/M2* | F25H5.3 | 6.30 | 60868 | 31 | 134 | 19 |  |  | 1.99 |  |
| 55 | NP_504700.2 | *Dihydrolipoamide succinyltransferase* | W02F12.5 | 9.08 | 49782 | 33 | 68 | 14 |  |  | 1.99 | 1.52 |
| **56** | NP_496801.2 | gaLECtin family member (lec-1) | lec-1 | 6.12 | 31790 | 51 | 145 | 19 |  |  | 1.81 | 1.9 |
| 57 | NP_001022405.1 | gaLECtin family member (lec-1) | lec-1 | 6.36 | 32664 | 17 | 60 | 5 |  | 1.19 | 1.3 | 1.71 |
| 58 | NP_001023185.1 | hypothetical protein F37C4.5 | F37C4.5 | 5.46 | 61577 | 56 | 182 | 25 |  |  | 1.74 | 1.35 |
| 59 | NP_509361.1 | *Tetrahydrofolate synthase* | K07E3.4 | 6.53 | 69368 | 40 | 180 | 26 |  |  | 1.72 |  |
| 60 | NP_499900.1 | *Purine nucleoside phosphorylase* | K02D7.1 | 5.62 | 32733 | 36 | 64 | 12 |  | 1.36 | 1.59 |  |
| 61 | NP_509584.1 | *Hydroxyacyl-coenzyme A dehydrogenase* | B0272.3 | 7.66 | 33530 | 60 | 131 | 17 |  |  | 1.51 | 1.37 |
| 62 | NP_001021221.1 | *Glutathione reductase* | C46F11.2 | 6.22 | 49999 | 45 | 115 | 20 | 1.23 |  | 1.49 |  |
| **63** | NP_001022078.1 | ALdehyde deHydrogenase family member (alh-8) | alh-8 | 7.56 | 56882 | 27 | 68 | 15 |  |  | 1.48 | 1.13 |
| 64 | NP_001021745.1 | *Dimethylglycine dehydrogenase* | Y37E3.17 | 7.06 | 92164 | 25 | 114 | 20 | 1.2 |  | 1.47 | 1.41 |
| 65 | NP_498642.1 | FUMarase family member (fum-1) | fum-1 | 7.77 | 53784 | 35 | 121 | 20 |  |  | 1.45 | 1.3 |
| **66** | NP_506260.1 | hypothetical protein W07G4.4 | W07G4.4 | 6.57 | 56510 | 32 | 110 | 15 |  |  | 1.42 |  |
| 67 | NP_509875.1 | *Isocitrate dehydrogenase* | idh-2 | 7.17 | 49470 | 45 | 118 | 21 |  |  | 1.4 | 1.18 |
| 68 | NP_506579.1 | *Dihydrolipoamide dehydrogenase* | F23B12.5 | 8.40 | 53719 | 42 | 104 | 18 |  |  | 1.37 | 1.2 |
| 69 | NP_496455.1 | *2-hydroxyacyl-CoA lyase 1* | B0334.3 | 8.74 | 69933 | 21 | 79 | 11 |  |  | 1.34 |  |
| 70 | NP_872253.1 | *Creatine kinase* | F46H5.3 | 6.17 | 40365 | 69 | 227 | 28 |  |  | 1.31 | 1.28 |
| 71 | NP_492330.2 | *Isocitrate dehydrogenase isoform 1* | F43G9.1 | 7.03 | 38898 | 72 | 210 | 26 |  |  | 1.3 | 1.29 |
| 72 | NP_498415.2 | LEThal family member (let-721) | let-721 | 6.27 | 65808 | 26 | 114 | 18 |  |  | 1.3 |  |
| 73 | NP_507877.1 | yeast SEC homolog family member (sec-23) | sec-23 | 6.81 | 90588 | 22 | 67 | 15 |  |  | 1.3 | 1.32 |
| 74 | NP_491245.1 | PhosphoGlycerate Kinase family member (pgk-1) | pgk-1 | 6.53 | 44199 | 35 | 119 | 20 |  |  | 1.28 | 1.16 |
| 75 | NP_499809.1 | ACTin family member (act-5) | act-5 | 5.44 | 42131 | 48 | 169 | 20 |  | 1.12 | 1.25 | 1.24 |
| 76 | NP_491926.1 | Lipid Binding Protein family member (lbp-6) | lbp-6 | 6.77 | 15631 | 86 | 140 | 15 |  | -2.52 | -2.87 |  |
| 77 | NP_509242.1 | *Aldose reductase* | C07D8.6 | 5.42 | 35283 | 61 | 183 | 22 |  |  | -2.5 |  |
| 78 | NP_504291.1 | Heat Shock Protein family member (hsp-6) | hsp-6 | 5.89 | 71086 | 41 | 143 | 25 |  |  | -2.37 | -1.6 |
| 79 | NP_872125.1 | hypothetical protein F17C11.9 | F17C11.9 | 5.30 | 42091 | 23 | 60 | 12 |  |  | -2.31 | -2.2 |
| 80 | NP_872125.1 | hypothetical protein F17C11.9 | F17C11.9 | 5.30 | 42091 | 22 | 107 | 14 |  |  | -1.34 | -1.51 |
| 81 | NP_498221.1 | Ribosomal Protein, Small subunit family member (rps-12) | rps-12 | 6.20 | 15459 | 71 | 89 | 10 |  | -1.51 | -2.16 | -1.63 |
| 82 | NP_498221.1 | Ribosomal Protein, Small subunit family member (rps-12) | rps-12 | 6.20 | 15459 | 68 | 71 | 9 |  | -1.63 | -1.41 |  |
| 83 | NP_504835.1 | FK506-Binding protein family member (fkb-3) | fkb-3 | 5.30 | 29347 | 50 | 91 | 14 |  | -1.56 | -2.03 | -1.6 |
| **84** | NP_506626.1 | abnormal DAuer Formation family member (daf-21) | daf-21 | 4.97 | 80689 | 37 | 142 | 28 |  |  | -1.8 | -1.57 |
| 85 | NP_499464.1 | DumPY : shorter than wild-type family member (dpy-18) | dpy-18 | 5.87 | 64172 | 31 | 92 | 21 |  |  | -1.68 | -1.43 |
| **86** | NP_001021051.1 | UDP-GALactose 4-Epimerase family member (gale-1) | gale-1 | 5.83 | 37944 | 46 | 84 | 12 |  |  | -1.67 |  |
| 87 | NP_001023756.1 | *Glutaredoxin-3* | D2063.3 | 5.13 | 38629 | 24 | 76 | 13 | -1.34 |  | -1.51 |  |
| 88 | NP_490843.1 | *Peptidase D* | K12C11.1 | 5.85 | 56045 | 22 | 96 | 16 |  | -1.31 | -1.48 | -1.26 |
| 89 | NP_871838.1 | *ATP-dependent RNA helicase DDX24* | F55F8.2 | 9.35 | 65928 | 25 | 70 | 18 |  |  | -1.48 |  |
| 90 | NP_504248.1 | Phosphoethanolamine MethyTransferase family member (pmt-2) | pmt-2 | 5.61 | 49909 | 42 | 149 | 21 |  |  | -1.44 | -2.05 |
| 91 | NP_504248.1 | Phosphoethanolamine MethyTransferase family member (pmt-2) | pmt-2 | 5.61 | 49909 | 45 | 139 | 22 |  |  | -1.27 |  |
| **92** | NP_494897.3 | *Maltase-glucoamylase* | R05F9.12 | 5.51 | 107287 | 31 | 150 | 28 |  |  | -1.43 | -2.39 |
| 93 | NP_502104.1 | *Isoform short of adenosine kinase* | R07H5.8 | 5.67 | 37917 | 37 | 84 | 11 |  |  | -1.39 |  |
| 94 | NP_497978.1 | Ribosomal Protein, Small subunit family member (rps-0) | rps-0 | 5.49 | 30800 | 48 | 129 | 11 |  | -1.09 | -1.38 | -1.35 |
| 95 | NP_001021695.1 | LEVamisole resistant family member (lev-11) | lev-11 | 4.66 | 32984 | 39 | 61 | 13 |  |  | -1.36 |  |
| 96 | NP_502080.1 | *Endoplasmin* | T05E11.3 | 4.97 | 87115 | 30 | 117 | 27 |  | -1.25 | -1.32 | -1.65 |
| 97 | NP_001022872.1 | *Phosphoglucomutase-2* | Y43F4B.5 | 5.82 | 66659 | 36 | 160 | 28 |  |  | -1.3 | -1.35 |
| 98 | NP_492765.1 | Proteasome Alpha Subunit family member (pas-5) | pas-5 | 5.27 | 27247 | 70 | 108 | 14 |  |  | -1.27 | -1.28 |
| 99 | NP_495137.1 | Intermediate Filament, B family member (ifb-1) | ifb-1 | 5.55 | 63775 | 22 | 77 | 14 |  |  | 2.46 | 5.37 |
| 100 | NP_495409.3 | hypothetical protein C44B7.10 | C44B7.10 | 8.70 | 50951 | 27 | 108 | 12 |  |  | 2.19 | 2.51 |
| 101 | NP_496090.1 | hypothetical protein ZK1320.9 | ZK1320.9 | 8.79 | 51622 | 18 | 68 | 9 |  |  |  | 1.96 |
| 102 | NP_508711.1 | Vacuolar H ATPase family member (vha-12) | vha-12 | 5.34 | 55006 | 35 | 165 | 14 |  |  | 1.19 | 1.65 |
| 103 | NP_501040.1 | Vacuolar H ATPase family member (vha-8) | vha-8 | 6.78 | 25742 | 54 | 141 | 16 |  |  |  | 1.57 |
| 104 | NP_506571.1 | Proteasome Alpha Subunit family member (pas-1) | pas-1 | 8.22 | 27260 | 42 | 102 | 12 |  |  |  | 1.52 |
| 105 | NP_741145.1 | Myosin Light Chain family member (mlc-3) | mlc-3 | 4.63 | 17134 | 83 | 191 | 16 |  |  |  | 1.5 |
| 106 | NP_506559.1 | Vacuolar H ATPase family member (vha-13) | vha-13 | 5.09 | 66874 | 30 | 104 | 18 |  |  |  | 1.49 |
| 107 | NP_497134.1 | *Phosphoenolpyruvate carboxykinase* | W05G11.6 | 6.03 | 73894 | 25 | 69 | 19 |  |  | 1.18 | 1.47 |
| 108 | NP_001022189.1 | gaLECtin family member (lec-2) | lec-2 | 6.19 | 31276 | 43 | 123 | 13 |  | 1.32 | 1.69 | 1.46 |
| 109 | NP_499264.1 | CiTrate Synthase family member (cts-1) | cts-1 | 7.68 | 51793 | 31 | 119 | 15 |  |  |  | 1.44 |
| 110 | NP_496868.1 | *Phosphoglycerate dehydrogenase* | C31C9.2 | 6.34 | 34841 | 51 | 151 | 15 |  |  | 2.17 | 1.43 |
| 111 | NP_509939.1 | Fourteen-Three-Three family member (ftt-2) | ftt-2 | 4.79 | 28164 | 30 | 96 | 10 |  |  |  | 1.43 |
| 112 | NP_001021529.1 | *ATP synthase subunit alpha* | H28O16.1 | 8.25 | 55100 | 49 | 231 | 25 |  |  |  | 1.37 |
| 113 | NP_509446.1 | Succinate DeHydrogenase complex subunit A family member (sdha-1) | sdha-1 | 6.37 | 70867 | 17 | 106 | 13 |  |  |  | 1.3 |
| 114 | NP_495494.1 | MAO-C-like dehydratase domain family member (maoc-1) | maoc-1 | 6.00 | 32929 | 28 | 59 | 7 |  |  |  | 1.3 |
| 115 | CAA34719.1 | actin | act3 | 5.30 | 42030 | 19 | 59 | 7 |  |  |  | 1.28 |
| 116 | NP_510011.1 | Ubiquinol-Cytochrome c oxidoReductase complex family member (ucr-2.1) | ucr-2.1 | 7.79 | 42799 | 27 | 65 | 9 |  |  |  | 1.26 |
| 117 | NP_492767.1 | TCTP (translationally-controlled tumor protein) homolog family member (tct-1) | tct-1 | 4.78 | 20643 | 71 | 89 | 10 |  | -1.8 |  | -2.03 |
| 118 | NP_496237.1 | GPD (glyceraldehyde 3-phosphate dehydrogenase) family member (gpd-1) | gpd-1 | 7.68 | 36531 | 46 | 105 | 13 |  |  | 1.13 | -1.78 |
| 119 | NP_509019.1 | Heat Shock Protein family member (hsp-3) | hsp-3 | 4.95 | 73095 | 19 | 74 | 10 |  |  |  | -1.73 |
| 120 | NP_508407.1 | hypothetical protein T24C12.4 | T24C12.4 | 8.92 | 25757 | 26 | 63 | 8 |  |  |  | -1.64 |
| 121 | NP_492360.1 | Proteasome Alpha Subunit family member (pas-4) | pas-4 | 5.93 | 28336 | 36 | 74 | 9 |  |  |  | -1.6 |
| 122 | NP_492457.1 | Elongation FacTor family member (eft-2) | eft-2 | 6.10 | 95477 | 13 | 63 | 12 |  |  |  | -1.48 |
| 123 | NP_501859.1 | RACK1 homolog family member (rack-1) | rack-1 | 6.44 | 36150 | 63 | 177 | 16 |  |  | -1.22 | -1.42 |
| 124 | NP_741154.1 | Chaperonin Containing TCP-1 family member (cct-6) | cct-6 | 6.27 | 48268 | 29 | 61 | 15 |  |  |  | -1.42 |
| 125 | NP_498868.1 | *Heat shock protein 105kDa isoform beta* | C30C11.4 | 5.31 | 87241 | 25 | 128 | 18 |  | -1.15 | -1.22 | -1.41 |
| 126 | NP_741144.1 | *46kDa protein* | F09F7.4 | 8.40 | 40402 | 26 | 92 | 12 |  |  |  | -1.39 |
| 127 | NP_499324.1 | 4-HydroxyPhenylpyruvate Dioxygenase (HPD) family member (hpd-1) | hpd-1 | 5.40 | 44640 | 47 | 136 | 17 |  |  |  | -1.33 |
| **128** | NP_491955.1 | *adenosylhomocysteinase* | ahcy-1 | 5.85 | 47905 | 27 | 130 | 13 |  |  |  | -1.3 |
| 129 | NP_503068.1 | Heat Shock Protein family member (hsp-1) | hsp-1 | 5.44 | 69965 | 23 | 80 | 16 |  |  |  | -1.29 |
| 130 | NP_504472.1 | Proteasome Alpha Subunit family member (pas-6) | pas-6 | 6.50 | 28350 | 70 | 143 | 13 |  |  |  | -1.25 |
