# Supplementary material for: Proteome changes of Caenorhabditis elegans upon a Staphylococcus aureus infection
Source: Biol Direct. 2010 Feb 17;5:11. doi: 10.1186/1745-6150-5-11 (PMC2834640; doi:10.1186/1745-6150-5-11)
Supplement: Additional file 2 — Comparison table (Table 3). Summary of proteins which are found differentially expressed upon challenge with both S. aureus (this paper) and A. hydrophila (Bogaerts et. al 2010, ref 29). "+" is up- and "-" is downregulated at least at one time point of the experiment. Proteins in red show an opposite pattern between the two conditions. [file 1745-6150-5-11-S2.DOC]

Table 3: **Summary of proteins which are found differentially expressed upon challenge with both *S. aureus* (this paper) and *A. hydrophila* (Bogaerts et al., 2010).** “+” is up- and “-“ is downregulated at least at one time point of the experiment. Proteins in red show an opposite pattern between the two conditions.

|  | ***A. hydrophila*** | ***S. aureus*** |
| --- | --- | --- |
| **Translation and ribosomal structures** |  |  |
| rpa-0 | + | + |
| **Chaperones** |  |  |
| daf-21 | - | - |
| cct-5 | - | - |
| **Metabolism** |  |  |
| sodh-1 | **-** | **+** |
| sodh-1 | **-** | **+** |
| **Carbohydrate transport and metabolism** |  |  |
| R05F9.12 | **+** | **-** |
| alh-8 | **-** | **+** |
| **Coenzyme transport and metabolism** |  |  |
| sams-1 | +/- | - |
| ahcy-1 | - | - |
| **Lipid transport and metabolism** |  |  |
| ech-6 | + | + |
| **Energy production and conversion** |  |  |
| R11A5.4 | - | - |
| R11A5.4 | - | - |
| **Cellular processes and signaling** |  |  |
| ifb-2 | - | -/+ |
| gale-1 | - | - |
| lec-1 | + | + |
| **Unknown** |  |  |
| W07G4.4 | + | + |
